# Gender-Specific Independent and Combined Effects of the Cortisol-to-Cortisone Ratio and 11-Deoxycortisol on Prediabetes and Type 2 Diabetes Mellitus: From the Henan Rural Cohort Study

**DOI:** 10.1155/2019/4693817

**Published:** 2019-06-02

**Authors:** Xue Liu, Jingjing Jiang, Xiaotian Liu, Zhicheng Luo, Yan Wang, Xiaokang Dong, Dandan Wei, Wenqian Huo, Songcheng Yu, Linlin Li, Shuna Jin, Chongjian Wang, Zhenxing Mao

**Affiliations:** ^1^Department of Epidemiology and Biostatistics, College of Public Health, Zhengzhou University, Zhengzhou, Henan, China; ^2^Department of Nutrition and Food Hygiene, College of Public Health, Zhengzhou University, Zhengzhou, Henan, China; ^3^Key Laboratory of Environment and Health, Ministry of Education & Ministry of Environmental Protection, and State Key Laboratory of Environmental Health, School of Public Health, Tongji Medical College, Huazhong University of Science and Technology, Wuhan, Hubei, China

## Abstract

**Objective:**

The aim of the study was to investigate the independent and combined effects of the cortisol-to-cortisone ratio (F/E) and 11-deoxycortisol on prediabetes and type 2 diabetes mellitus (T2DM) among different genders.

**Methods:**

A case-control study was performed including 2676 participants from the Henan Rural Cohort Study. Liquid chromatography-tandem mass spectrometry was used to assess serum cortisol, cortisone, and 11-deoxycortisol. Conditional logistic regression was performed to estimate the associations between hormones and outcomes.

**Results:**

After adjusting for multiple variables, the negative associations of F/E and 11-dexyocortisol with T2DM were observed in females (T3 vs. T1: OR = 0.56, 95% CI: 0.39-0.80 for F/E; T3 vs. T1: OR = 0.44, 95% CI: 0.27-0.73 for 11-dexyocortisol). However, only 11-dexyocortisol showed a negative association with prediabetes both in males and females. Compared with the combination of low F/E and 11-dexyocortisol, the combination of middle F/E and high 11-dexyocortisol was significantly associated with prediabetes (OR = 0.29, 95% CI: 0.12-0.71) in males. Furthermore, the combination of high F/E and 11-dexyocortisol was associated with the lowest odds of prediabetes (OR = 0.39, 95% CI: 0.21-0.73) and T2DM (OR = 0.25, 95% CI: 0.12-0.52) in females.

**Conclusions:**

Serum F/E level was negatively associated with T2DM only in females whereas serum 11-deoxycortisol level was negatively associated with prediabetes in males and with prediabetes and T2DM in females. Additionally, their combination has a synergistic effect on T2DM.

## 1. Introduction

The prevalence of diabetes is increasing rapidly in parallel with the economy and urbanization in China. By 2013, the prevalence of diabetes and prediabetes among Chinese adults was about 10.9% and 35.7%, respectively. Furthermore, prediabetes was more prevalent in rural residents than in urban residents [1–4]. Mounting evidence points to prediabetes conditions as risk factors for overt diabetes [5–7]. In addition to excessive food intake and sedentary lifestyles, it is increasingly recognized that genetic and hormonal homeostasis imbalances can also contribute to diabetes [8].

Glucocorticoid (GC) and its intracellular receptor, glucocorticoid receptor (GR), are key checkpoints for endocrine control of energy balance in mammals [9, 10]. Lack or excess of endogenous or exogenous GC can disturb the energy metabolism of the whole body, especially concerning the handling pathways of glucose and lipid [11–13]. Cortisol is a kind of glucocorticoid which is produced by the 11-deoxycortisol in the adrenal cortical mitochondria. It is a key element of the end effector of the hypothalamic-pituitary-adrenal (HPA) axis and regulated by 11*β*-hydroxysteroid dehydrogenase type 1 (11*β*-HSD1) and 11*β*-hydroxysteroid dehydrogenase type 2 (11*β*-HSD2) [14, 15]. 11*β*-HSD2 acts principally as a dehydrogenase to convert cortisol into cortisone. In contrast to 11*β*-HSD2, 11*β*-HSD1 works predominantly in the opposite direction, reducing cortisone to cortisol in the liver, adipose, and other tissues [16, 17]. Because of the complex interactions of these two isoenzymes that affect cortisol systemic metabolism, the contribution of 11*β*-HSD to diabetes remains unclear. 11-Dexyocortisol is an intermediate product of the transformation of cholesterol into cortisol. Plasma 11-dexyocortisol concentration increased significantly in patients with congenital adrenal hyperplasia, which indicates that 11-dexyocortisol plays an important role in physiological activity.

Previous studies have suggested that salivary cortisol levels are associated with type 2 diabetes mellitus (T2DM), and the serum cortisol-to-cortisone ratio (F/E) could serve as a marker of net 11*β*-HSD activity [18–21]. Moreover, a few animal experiments have shown that GCs can inhibit insulin secretion in vitro [22, 23]. Meanwhile, there are epidemiological studies investigating the relationship between F/E and T2DM and prediabetes [19, 24, 25]. Previous research has evidenced the impact of gender differences in the relationship between cortisol and diabetes [26]. However, no epidemiological researches on the association between 11-dexyocortisol and glucose metabolism were reported.

In summary, we aim to examine (1) the effect of F/E and 11-deoxycortisol on prediabetes and T2DM, (2) the potential impact of gender on this association, and (3) combined effects of F/E and 11-deoxycortisol on prediabetes and T2DM among rural residents in Henan Province, China.

## 2. Materials and Methods

### 2.1. Study Population

The participants of this study were from the Henan Rural Cohort Study, with the aim of identifying environmental and genetic risk factors (and their interactions) that contribute to the development of major chronic disease, such as T2DM, hypertension, and the metabolic syndrome [27].

According to the inclusion criteria, we excluded participants who received sex steroids or other androgen-related drugs. 925 individuals with T2DM who were eligible to participate were selected. Normal glucose tolerance (NGT) and prediabetes patients were matched to T2DM cases by age (in 3-year strata) and sex. Participants who were without information about the steroid hormone (*n* = 9), had extreme cortisol values (*n* = 48) or cortisone values (*n* = 6), and were without matched cases or controls (*n* = 36) were excluded. Finally, a total of 2676 participants were included in this analysis.

The protocol of this study was in accordance with the guidelines of the 1964 Helsinki Declaration, and the ethic committee of the Zhengzhou University Life Science Ethics Committee (Code: [2015] MEC (S128)) approved this study. Written informed consent was obtained from all individual participants included in the study.

### 2.2. Data Collection

Information on the general characteristics of participants' demography (age, gender, educational attainment, socioeconomic status, etc.), lifestyles (smoking status, alcohol intake, physical activity, etc.), and individual and family histories of disease was obtained by well-trained investigators through face-to-face interviews using a structured questionnaire. Height, weight and waist circumference were measured twice. The blood pressure was measured three times with a 30-second interval between each measurement in sitting position, and the mean value was obtained.

The educational attainment classification is bounded by middle school. Socioeconomic status was evaluated based on per capita monthly income (<500, ~500, and ≥1000 renminbi (RMB)). Smoking status was defined as smoking at least one cigarette per day for six consecutive months. Alcohol intake was defined as drinking alcohol at least 12 times per year. Physical activity was categorized into low, mediate, and high level according to the international physical activity questionnaire-short (IPAQ-short). Body mass index (BMI) was calculated as weight divided by square of height (kg/m^2^).

### 2.3. Laboratory Measurements

Venous blood samples were collected for biochemical analysis after fasting for at least 8 h, then the serum after centrifugation were stored in a -80°C freezer before analysis. All biochemical indicators, including fasting plasma glucose (FPG), triglycerides (TC), triglyceride (TG), high-density lipoprotein cholesterol (HDL-C), and low-density lipoprotein cholesterol (LDL-C), were measured by the ROCHE Cobas C501 automatic biochemical analyzer. Glycosylated hemoglobin (HbA1c) was determined enzymatically on an automatic analyzer.

Concentration of cortisol, cortisone, and 11-deoxycortisol was measured using liquid chromatography-tandem mass spectrometry on a Waters XEVO TQ-S system (Waters, Milford, MA, USA) [24, 28]. Glucocorticoid was quantitated using the internal reference in the positive ion mode with multiple reaction monitoring. After internal standard recovery correction, the peaks were integrated, and the concentration was determined from a standard curve and peak area. Values below the quantification limit or undetected were replaced by half of the detection limit. Cases and matched controls were measured in the same analytical run, but we did not know whether the sample was a case or a control in the test (blind determination).

### 2.4. Ascertainment of Cases

The diagnostic criteria for prediabetes and T2DM were recommended by the World Health Organization (WHO) (1999) and American Diabetes Association (ADA) (2002) guidelines. Prediabetes was defined as 6.1 mmol/L ≤ FPG < 7.0 mmol/L or 7% < HbA1C < 6.5% while T2DM was defined as FPG ≥ 7.0 mmol/L or HbA1C ≥ 6.5% or having a self-reported previous diagnosis of diabetes by a physician and on antiglycemic agents during the previous 2 weeks, after excluding type 1 diabetes, gestational diabetes, and other special types of diabetes at the same time.

### 2.5. Statistical Analysis

Continuous variables were presented as the means ± standard deviation (SD) or median (interquartile range) while the categorical data was given as frequencies (%). The statistical differences between groups were analyzed using a two-sample paired *t*-test (continuous variables) or chi-square tests (categorical variables). Because of skewed distribution of serum F/E and 11-dexyocortisol levels, these variables were natural log-transformed before analysis and defined as ln-F/E and ln-11-dexyocortisol. Serum F/E and 11-dexyocortisol concentrations were categorized in tertiles in males and females, respectively. Conditional logistic regression was used to estimate the associations of hormones with prediabetes and T2DM. Odds ratios (ORs) and 95% confidence intervals (CIs) were reported. To study the relationship between the increasing tertiles and prediabetes and T2DM, trend tests were performed by putting the categorical variables as continuous variables in regression analysis. Multivariable adjustment modelling was performed:

Model 1: no adjustment
Model 2: BMI, smoking status, alcohol intake, physical activity, per capita monthly income, educational attainment, and family history of T2DM (BMI and WC were highly correlated (Pearson correlation coefficient = 0.795, *P* < 0.001), so only BMI was used as an indicator of adiposity)
Model 3: as for model 2, plus SBP, TC, TG, HDL-C, and LDL-C

The combined effects of F/E and 11-dexyocortisol on prediabetes and T2DM were tested by including the terms of the corresponding tertiles in conditional logistic regression analysis based on model 3 [29, 30]. Multiple imputation procedure was used to adjust for potential bias caused by missing data (*n* = 12 imputations) [31]. All analyses were performed using SPSS statistical software (SPSS, version 19.0; SPSS Inc., Chicago, IL, USA). Two-tailed *P* < 0.05 was considered significant.

## 3. Results

### 3.1. Characteristics of Participants

A total of 2676 participants (mean age = 59.77 years, males = 1027) were enrolled in this study, including 906 NGT, 878 prediabetes, and 892 T2DM. The basic characteristics of the NGT and prediabetes participants are presented in Table 1. Compared with NGT participants, prediabetes patients had higher BMI, SBP, TC, TG, and LDL-C and lower HDL-C in males. In females, prediabetes patients were more likely to have higher BMI, TC, TG, and LDL-C than NGT participants.  Table 2 summarizes the basic characteristics of the NGT and T2DM participants.

In males, patients with T2DM were more likely to have higher BMI, SBP, TC, and TG and more likely to be a current smoker and have family history of T2DM than NGT participants. Meanwhile, T2DM patients also tended to have lower HDL-C and educational attainment. In females, compared with NGT participants, T2DM patients had higher BMI, SBP, TC, TG, and LDL-C and more family history of T2DM. However, patients with T2DM also tended to have lower HDL-C and physical activity levels.

### 3.2. The Separate Associations of F/E and 11-Dexyocortisol with Prediabetes and T2DM

As shown in Table 3, the negative association of 11-dexyocortisol with prediabetes in males was observed. The finding remained significant after adjusting multiple variables in model 3. Per 1 unit increase in ln-11-deoxycortisol was negatively associated with prediabetes (OR = 0.76, 95%CI = 0.61‐0.93). The third tertile of ln-11-deoxycortisol was also negatively associated with prediabetes (OR = 0.40, 95%CI = 0.22‐0.71) versus tertile 1. No significant associations between ln-F/E and prediabetes and T2DM were observed in males after adjusting multiple variables.

In females, 11-dexyocortisol showed a negative association with prediabetes while per 1 unit increase in ln-11-deoxycortisol was negatively associated with prediabetes (OR = 0.70, 95%CI = 0.59‐0.83). The second and third tertiles of ln-11-deoxycortisol were also negatively associated with prediabetes (OR = 0.43, 95%CI = 0.28‐0.66; OR = 0.40, 95%CI = 0.26‐0.62, respectively) versus tertile 1. Per 1 unit increase in 11-dexyocortisol was also significantly and negatively associated with T2DM (OR = 0.78, 95%CI = 0.65‐0.93). The second and third tertiles of ln-11-deoxycortisol were also negatively associated with T2DM (OR = 0.48, 95%CI = 0.30‐0.77; OR = 0.44, 95%CI = 0.27‐0.73, respectively) versus tertile 1. The association of increased ln-F/E with T2DM reached significance in females (per 1 unit increase in ln-F/E, OR = 0.72, 95%CI = 0.62‐0.83). For T2DM in females, the adjusted ORs for second and third tertiles of ln-F/E were 0.62 (95%CI = 0.44‐0.88) and 0.56 (95%CI = 0.39‐0.80), respectively.

### 3.3. The Combined Effect of F/E and 11-Dexyocortisol on Prediabetes and T2DM

**Figure 1:**
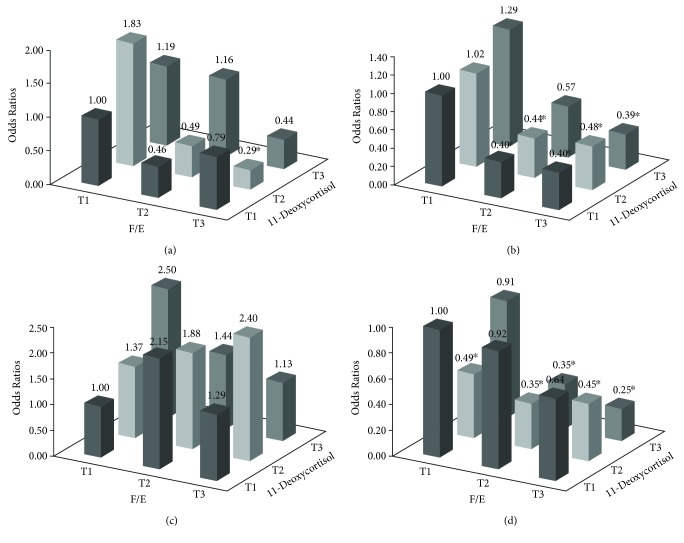
The effect of F/E (cortisol-to-cortisone ratio) and 11-deoxycortisol combined on prediabetes and type 2 diabetes mellitus (T2DM): (a) male prediabetes; (b) female prediabetes; (c) male T2DM; (d) female T2DM. T: tertiles. ^∗^*P* < 0.05.

As shown in Figure 1, the combination of middle F/E and high 11-dexyocortisol was significantly associated with prediabetes (OR = 0.29, 95% CI: 0.12-0.71) in males. In females, the combined effects of F/E and 11-dexyocortisol on prediabetes and T2DM were observed. There was a significant enhancement effect between F/E and 11-dexyocortisol. Compared with the combination of low F/E and 11-dexyocortisol, most groups trended toward lower odds of prediabetes and T2DM, but the combination of highest F/E and 11-dexyocortisol was associated with the lowest odds of prediabetes (OR = 0.39, 95% CI: 0.21-0.73) and T2DM (OR = 0.25, 95% CI: 0.12-0.52) based on model 3.

## 4. Discussion

In this case-control study, we explored the effect of F/E and 11-dexyocortisol on prediabetes and T2DM. We found that F/E levels were negatively associated with the T2DM in females independent of established diabetes risk factors including BMI, alcohol drinking, SBP, and TC. However, there was no association between F/E and prediabetes. The association between the 11-deoxycortisol level and T2DM was significant only in females. However, the inverse association between 11-deoxycortisol and prediabetes was prominent both in males and females. Furthermore, their combination had a synergistic effect on prediabetes and T2DM in females.

Several epidemiological studies have previously assessed the relationship between F/E and the risk of T2DM. A nested case-control study included 3000 subjects; both in univariable and in fully adjusted multivariable survival regressions, F/E quartiles are negatively associated with blood glucose levels, which is consistent with our finding [19]. However, our study found that only in females did F/E correlate with T2DM [32], which was contrary to another finding from the cross-sectional population-based KORA-age study [18]. We had not found evidence to explain that, so it could be a biomarker to distinguish T2DM in females. We also explored the relationship between F/E and prediabetes, although no significant association was found. This study found a negative association between 11-deoxycortisol and prediabetes, which was not altered by gender. Thus, our findings suggested that F/E represent independent candidate predictors, will provide new insights into disease pathophysiology, and are potential targets for treatment.

Cortisol is an essential glucocorticoid and regulated by 11*β*-HSD1 and 11*β*-HSD2. Because of the complex interactions of these two isoenzymes that affect cortisol systemic metabolism, the contribution of 11*β*-HSD to diabetes remains unclear [33, 34]. Diabetic and chronic renal failure patients with hypertension have been reported to have abnormal ratios and higher complication rates. In this study, F/E levels were negatively associated with T2DM only in females. It might serve as a biomarker to predicting T2DM in females. 11-Deoxycortisol is an intermediate product in the conversion of cholesterol to cortisol, but how it regulates glucose metabolism is still not clear [21, 35, 36]. It may also serve as a biomarker for estimating the level of cortisol metabolism, but its serum content is very low compared with cortisol levels [37, 38]. Therefore, compared with F/E, 11-deoxycortisol is less effective as a biomarker to distinguish diabetes. However, in our study, 11-deoxycortisol was specifically associated with prediabetes in males, so 11-deoxycortisol has a potential to distinguish prediabetes independently of F/E. We hypothesized that the activation of cortisol conversion and the weakening of 11-deoxycortisol to cortisol contribute to the metabolism and regulation of blood glucose. The specific mechanism underlying the effects of 11-deoxycortisol on the development of diabetes also requires extensive animal experiments and epidemiological studies. However, animal data should be carefully converted to human physiology, as humans may have more pronounced hormonal regulation and biosynthetic mode.

The main strength of the present study is that we included prediabetes patients in this study to explore the relationship between F/E and 11-deoxycortisol and prediabetes. Moreover, we examined not only the relationship between 11-deoxycortisol and the outcome but also the combined effects of F/E and 11-deoxycortisol. To our knowledge, the present study is the first to suggest that the combined index of pronounced combination of high F/E and 11-deoxycortisol concentrations has a combined detrimental effect on the risk of developing T2DM and prediabetes in females. However, our study also has some limitations. First, even though we have adjusted the traditional diabetes risk factors, the possibility of residual confounders remains. Second, endogenous glucocorticoid secretion has circadian rhythm, lowest at midnight and highest in the morning. Because fasting venous blood in the morning were collected, the estimates of hormone levels may be skewed. Third, we did not rule out participants using glucocorticoid replacement therapy, although they were rare in the study. Last but not least, serum F/E levels instead of F/E in urine were measured to represent the 11*β*-HSD activity, which may not be appropriate and accurate. However, the measurements of F/E in urine require interstitial measurements which are not suitable to study a large number of subjects.

In conclusion, serum F/E and 11-deoxycortisol levels were both negatively associated with prediabetes and T2DM. Their combination had a synergistic effect on distinguishing prediabetes and T2DM in females. Furthermore, F/E was an effective and independent marker for T2DM in females. Extensive animal experiments and epidemiological studies are needed to confirm this relationship and explore the underlying mechanisms.

## Figures and Tables

**Table 1:** Basic characteristics of the NGT and prediabetes participants.

	Male			Female		
	Prediabetes	NGT	*P* value	Prediabetes	NGT	*P* value
Subjects, *n*	342	342		536	536	
Age (years)	59.92 ± 8.94	59.92 ± 8.91	Matched	59.66 ± 8.62	59.68 ± 8.63	Matched
BMI (kg/m^2^)	24.14 ± 3.28	23.17 ± 3.10	<0.001	24.52 ± 3.47	23.87 ± 3.42	0.002
Current smoker, *n* (%)	158 (46.2)	174 (50.9)	0.221	0	2 (0.4)	0.25
Alcohol drinker, *n* (%)	84 (24.6)	102 (29.8)	0.122	10 (1.9)	12 (2.2)	0.667
Physical activity, *n* (%)			0.465			0.866
Low	119 (34.8)	104 (30.4)		110 (20.5)	103 (19.2)	
Mediate	119 (34.8)	129 (37.7)		308 (57.5)	313 (58.4)	
High	104 (30.4)	109 (31.9)		118 (22.0)	120 (22.4)	
Middle school or below, *n* (%)	45 (13.2)	43 (12.6)	0.819	33 (6.2)	26 (4.9)	0.349
Per capita monthly income, *n* (%)			0.076			0.563
<500 (RMB)	151 (44.2)	128 (37.4)		211 (39.4)	214 (39.9)	
~500 (RMB)	107 (31.3)	105 (30.7)		182 (34.0)	167 (31.2)	
≥1000 (RMB)	84 (24.6)	109 (31.9)		143 (26.7)	155 (28.9)	
Marital status, *n* (%)			0.605			0.219
Married/cohabiting	303 (88.6)	305 (89.2)		472 (88.1)	473 (88.2)	
Widowed	23 (6.7)	23 (6.7)		61 (11.4)	63 (11.8)	
Divorced/separated	4 (1.2)	1 (0.3)		3 (0.6)	0	
Unmarried	12 (3.5)	13 (3.8)		0	0	
Family history of T2DM, *n* (%)	5 (1.5)	5 (1.5)	0.624	8 (1.5)	9 (1.7)	0.500
SBP (mmHg)	121.69 ± 15.8	118.74 ± 16.12	0.001	124.93 ± 20.03	121.90 ± 19.28	0.072
TC (mmol/L)	4.68 ± 0.84	4.42 ± 0.73	<0.001	4.99 ± 0.88	4.72 ± 0.84	<0.001
TG (mmol/L)	1.81 ± 1.13	1.59 ± 1.10	0.001	1.89 ± 1.07	1.72 ± 1.03	0.048
HDL-C (mmol/L)	1.29 ± 0.34	1.34 ± 0.34	0.035	1.40 ± 0.33	1.43 ± 0.34	0.151
LDL-C (mmol/L)	2.90 ± 0.75	2.72 ± 0.66	<0.001	3.10 ± 0.83	2.89 ± 0.74	<0.001
F/E	11.62 (8.10-16.62)	11.25 (8.44-15.54)	0.345	11.33 (8.15-16.29)	11.41 (8.50-15.86)	0.561
11-Deoxycortisol (ng/mL)	0.30 (0.20-0.50)	0.40 (0.20-0.60)	0.001	0.30 (0.20-0.50)	0.30 (0.20-0.50)	0.028

Abbreviation: NGT: normal glucose tolerance; T2DM: type 2 diabetes mellitus; BMI: body mass index; RMB: renminbi; SBP: systolic blood pressure; TC: total cholesterol; TG: triacylglycerol; HDL-C: high-density lipoprotein cholesterol; LDL-C: low-density lipoprotein cholesterol; F/E: cortisol-to-cortisone ratio; SD: standard deviation. Data are presented as *n* (%) for categorical data and mean ± SD or median (interquartile range) for continuous data.

**Table 2:** Basic characteristics of the NGT and T2DM participants.

	Male			Female		
	T2DM	NGT	*P* value	T2DM	NGT	*P* value
Subjects, *n*	339	339		553	553	
Age (years)	59.92 ± 8.91	59.92 ± 8.91	Matched	59.66 ± 8.61	59.66 ± 8.60	Matched
BMI (kg/m^2^)	25.30 ± 3.32	23.12 ± 3.07	<0.001	25.68 ± 3.63	23.81 ± 3.43	<0.001
Current smoker, *n* (%)	144 (42.5)	173 (51.0)	0.026	2 (0.4)	3 (0.5)	0.687
Alcohol drinker, *n* (%)	113 (33.3)	102 (30.1)	0.364	7 (1.3)	12 (2.2)	0.247
Physical activity, *n* (%)			0.197			0.048
Low	114 (33.6)	100 (29.5)		139 (25.1)	105 (19.0)	
Mediate	107 (31.6)	129 (38.1)		300 (54.2)	325 (58.8)	
High	118 (34.8)	110 (32.4)		114 (20.6)	123 (22.2)	
Middle school or below, *n* (%)	271 (79.9)	298 (87.9)	0.003	526 (95.1)	526 (95.1)	0.555
Per capita monthly income, *n* (%)			0.439			0.748
<500 (RMB)	137 (40.4)	128 (37.8)		226 (40.9)	223 (40.3)	
~500 (RMB)	88 (26.0)	103 (30.4)		162 (29.3)	173 (31.3)	
≥1000 (RMB)	114 (33.6)	108 (31.9)		165 (29.8)	157 (28.4)	
Marital status, *n* (%)			0.760			0.730
Married/cohabiting	298 (87.9)	302 (89.1)		495 (89.5)	489 (88.5)	
Widowed	23 (6.8)	23 (6.8)		54 (9.8)	61 (11.0)	
Divorced/separated	3 (0.9)	1 (0.3)		4 (0.7)	3 (0.5)	
Unmarried	15 (4.4)	13 (93.8)		0	0	
Family history of T2DM, *n* (%)	17 (5.0)	5 (1.5)	0.009	32 (5.8)	10 (1.8)	0.001
SBP (mmHg)	123.59 ± 16.57	118.71 ± 16.26	0.001	128.93 ± 18.59	121.57 ± 19.17	<0.001
TC (mmol/L)	4.65 ± 1.02	4.41 ± 0.73	<0.001	5.07 ± 1.09	4.71 ± 0.84	<0.001
TG (mmol/L)	2.30 ± 1.62	1.59 ± 1.10	0.001	2.41 ± 1.51	1.70 ± 1.01	<0.001
HDL-C (mmol/L)	1.19 ± 0.32	1.34 ± 0.34	0.001	1.32 ± 0.33	1.43 ± 0.34	<0.001
LDL-C (mmol/L)	2.68 ± 0.89	2.71 ± 0.66	0.576	2.98 ± 0.96	2.87 ± 0.74	0.047
F/E	11.17 (8.13-15.58)	11.33 (8.46-15.78)	0.296	10.28 (7.30-15.48)	11.45 (8.51-15.76)	0.967
11-Deoxycortisol (ng/mL)	0.30 (0.20-0.50)	0.20 (0.30-0.60)	0.572	0.30 (0.20-0.50)	0.30 (0.20-0.50)	0.649

Abbreviation: NGT: normal glucose tolerance; T2DM: type 2 diabetes mellitus; BMI: body mass index; RMB: renminbi; SBP: systolic blood pressure; TC: total cholesterol; TG: triacylglycerol; HDL-C: high-density lipoprotein cholesterol; LDL-C: low-density lipoprotein cholesterol; F/E: cortisol-to-cortisone ratio; SD: standard deviation. Data are presented as *n* (%) for categorical data and mean ± SD or median (interquartile range) for continuous data.

**Table 3:** Associations of ln-F/E and ln-11-deoxycortisol with prediabetes and T2DM in a male and female.

	Male			Female		
	Model 1	Model 2	Model 3	Model 1	Model 2	Model 3
*Prediabetes*						
Ln-F/E						
Continuous	0.91 (0.78-1.07)	0.92 (0.78-1.08)	0.93 (0.78-1.10)	0.88 (0.77-1.01)	0.90 (0.78-1.03)	0.90 (0.78-1.03)
T1	Reference	Reference	Reference	Reference	Reference	Reference
T2	**0.67 (0.46-0.96)**	0.73 (0.50-1.06)	0.71 (0.48-1.06)	0.98 (0.72-1.32)	1.01 (0.74-1.37)	1.07 (0.78-1.47)
T3	0.97 (0.66-1.43)	1.07 (0.83-1.84)	1.07 (0.70-1.64)	0.97 (0.73-1.30)	1.04 (0.77-1.40)	1.06 (0.77-1.45)
Ln-11-deoxycortisol						
Continuous	**0.73 (0.60-0.89)**	**0.75 (0.61-0.92)**	**0.76 (0.61-0.93)**	**0.73 (0.62-0.86)**	**0.74 (0.63-0.87)**	**0.70 (0.59-0.83)**
T1	Reference	Reference	Reference	Reference	Reference	Reference
T2	0.69 (0.40-1.19)	0.57 (0.32-1.01)	0.58 (0.32-1.05)	**0.43 (0.29-0.64)**	**0.44 (0.29-0.67)**	**0.43 (0.28-0.66)**
T3	**0.43 (0.25-0.73)**	**0.40 (0.23-0.70)**	**0.40 (0.22-0.71)**	**0.42 (0.28-0.63)**	**0.42 (0.28-0.65)**	**0.40 (0.26-0.62)**
*T2DM*						
Ln-F/E						
Continuous	0.92 (0.81-1.06)	0.92 (0.80-1.07)	0.88 (0.76-1.03)	**0.79 (0.69-0.90)**	**0.77 (0.70-0.88)**	**0.72 (0.62-0.83)**
T1	Reference	Reference	Reference	Reference	Reference	Reference
T2	0.79 (0.55-1.13)	0.90 (0.59-1.39)	0.97 (0.61-1.53)	**0.67 (0.50-0.90)**	**0.67 (0.49-0.93)**	**0.62 (0.44-0.88)**
T3	0.84 (0.58-1.22)	1.01 (0.66-1.55)	0.86 (0.54-1.36)	**0.61 (0.45-0.83)**	**0.65 (0.47-0.90)**	**0.56 (0.39-0.80)**
Ln-11-deoxycortisol						
Continuous	0.97 (0.79-1.19)	1.03 (0.82-1.30)	1.57 (0.87-2.81)	0.86 (0.74-1.01)	0.85 (0.72-1.01)	**0.78 (0.65-0.93)**
T1	Reference	Reference	Reference	Reference	Reference	Reference
T2	1.13 (0.64-2.01)	1.18 (0.61-2.27)	1.23 (0.61-2.48)	**0.55 (0.37-0.82)**	**0.51 (0.33-0.79)**	**0.48 (0.30-0.77)**
T3	0.88 (0.50-1.57)	1.02 (0.53-1.97)	1.03 (0.51-2.09)	**0.58 (0.38-0.88)**	**0.53 (0.34-0.85)**	**0.44 (0.27-0.73)**

Data are ORs (95% CI). Abbreviation: ln-: natural log-transformed; F/E: cortisol-to-cortisone ratio; T2DM: type 2 diabetes mellitus; ORs: odds ratios; CI: confidence interval. Model 1: no adjustment; model 2: adjusted for BMI, smoking status, alcohol intake, physical activity, per capita monthly income, educational attainment, and family history of T2DM; model 3: model 2+SBP, TC, TG, HDL-C, and LDL-C. Boldface values: *P* < 0.05.

## Data Availability

The datasets generated during and/or analyzed during the current study are available from the corresponding author on reasonable request.
